# Characterization and Therapeutic Use of Extracellular Vesicles Derived from Platelets

**DOI:** 10.3390/ijms22189701

**Published:** 2021-09-08

**Authors:** Timea Spakova, Jana Janockova, Jan Rosocha

**Affiliations:** Associated Tissue Bank of Faculty of Medicine of P. J. Safarik University and L. Pasteur University Hospital, SK-040 11 Kosice, Slovakia; jana.janockova@upjs.sk (J.J.); jan.rosocha@upjs.sk (J.R.)

**Keywords:** platelet, platelet-rich plasma, platelet-derived extracellular vesicles, platelet-derived exosomes, therapeutic application

## Abstract

Autologous blood products, such as platelet-rich plasma (PRP), are gaining increasing interest in different fields of regenerative medicine. Although growth factors, the main components of PRP, are thought to stimulate reparation processes, the exact mechanism of action and main effectors of PRP are not fully understood. Plasma contains a high amount of extracellular vesicles (EVs) produced by different cells, including anucleated platelets. Platelet-derived EVs (PL-EVs) are the most abundant type of EVs in circulation. Numerous advantages of PL-EVs, including their ability to be released locally, their ease of travel through the body, their low immunogenicity and tumourigenicity, the modulation of signal transduction as well as the ease with which they can be obtained, has attracted increased attention n. This review focuses briefly on the biological characteristics and isolation methods of PL-EVs, including exosomes derived from platelets (PL-EXOs), and their involvement in the pathology of diseases. Evidence that shows how PL-EVs can be used as a novel tool in medicine, particularly in therapeutic and regenerative medicine, is also discussed in this review.

## 1. Introduction

Blood is composed of different cellular, sub-cellular and molecular components that are involved in essential stages of wound healing [1,2] and regenerative processes [3,4]. Autologous blood products are subfractions of whole blood of a patient and are produced for direct application in ambulatory treatment. Notably, platelet-rich plasma (PRP) represents a well-known autologous derivative of whole blood with favourable immune, haemostatic and regenerative effects [5] and is characterized by a higher than baseline concentration of platelets prepared by centrifugal separation. It has been used in various medical fields, mainly including orthopaedics [6,7,8] as well as sport medicine [9,10], soft tissue injuries [11,12], dentistry [13,14], dermatology [15,16] and pain management [6].

Platelets, also named thrombocytes, are disc-shaped irregular components of blood and are essential for central physiological processes. Although platelets are classified as the smallest cells of blood without a nucleus, they exhibit active RNA metabolism during their relatively short life (~7-day half-life) [17]. They are derived from large megakaryocytes in bone marrow during thrombopoiesis. Although the primary function of platelets is haemostasis, they also contain and transport molecules implicated in numerous physiological and regenerative processes, mainly including wound healing [18], cell activation and proliferation [19,20], angiogenesis [21], immune cell recruitment and inflammation [22], bone regeneration [23] and cartilage repair [8]. It is believed that activated platelets secrete high amounts of growth factors (GFs), immunoglobulins, cytokines and molecules that actively contribute to the tissue-repairing process and support key functions of PRP. The most important natural growth factors—platelet-derived growth factor (PDGF), transforming growth factor (TGF), epidermal growth factor (EGF), insulin-like growth factor (IGF), basic fibroblast growth factor (FGF) and vascular endothelial growth factor (VEGF)—found in the α-granules of platelets stimulate cell proliferation, migration, chemotaxis, differentiation and angiogenesis, and promote the formation of extracellular matrix. Although PRP has broad prospects for therapeutic use, its specific molecular mechanism is still unclear. Beside growth factors, blood products contain a heterogeneous group of cell-derived membrane vesicles, called extracellular vesicles (EVs), which may be the main contributors to PRP effects. Plasma-derived EVs may originate from different cell types, such as leucocytes, erythrocytes, dendritic cells (DCs), platelets, mast cells, epithelial cells, endothelial cells and neurons. In fact, the majority (about 25%) of blood-derived EVs are thought to originate from the megakaryocytes, i.e., either from circulating platelets or directly from platelet precursor cells, which reside in the bone marrow [24,25]. EVs released from activated platelets can be distinguished from those generated from megakaryocytes the by expression of typical activation markers, such as P-selectin (CD62P). In 1967, platelet-derived EVs (PL-EVs) were observed for the first time by Wolf with the help of electron microscopy [26]. He named these lipid-rich particles “platelet dust” with procoagulant activity. Based on these observations, Warren et al. further demonstrated that PL-EVs were released during the adhesion of platelets to the subendothelial layer of coronary arteries in men [27].

It has been found that upon activation, blood platelets are able to release two main types of EVs into the bloodstream, i.e., microparticles (MPs) and exosomes (EXOs) [28], which can be recognised by different target cells such as endothelial cells and monocytes [29,30]. A recent study by McArthur et al. demonstrated that platelets may also release apoptotic bodies (1000–3000 nm) due to them undergoing apoptosis [31]. Platelet-derived microparticles (PL-MPs) are plasma membrane-derived larger vesicles (100 nm–1 μm) released from cells during stress conditions, including activation and apoptosis, with a typical immunophenotype of platelets and megakaryocytes [32]. PL-MPs are more likely to contain protein cargo of the mother cell and they express and transfer functional receptors from platelet membranes to other cell types [33,34]. Platelet-derived exosomes (PL-EXOs) with sub-micrometre diameters (30–100 nm) come from multivesicular bodies and α-granules by the endocytic pathway and have been shown to be highly modulated by the environmental conditions. Therefore, the exosome composition is more variable and seems to reflect the physiological status of the secretory cell. PL-EXOs include proteins, mRNAs and miRNAs [35]. An overlap of both subtypes of PL-EVs is apparent and is affected mainly by the isolation protocol and detection technique, and often leads to the inability to distinguish between them. Nevertheless, PL-MPs often expose phosphatidylserine [36], whereas the marker of platelet origin, CD41, [37,38] and higher amounts of CD63 were found in PL-EXOs [28].

PL-EVs were mainly studied for their role in blood coagulation and have proven their thrombogenic properties [39,40]. Recent evidence shows that PL-EVs act as a cargo of several functional molecules, including signalling mediators, growth factors, lipids, proteins, nucleic acids (e.g., circRNA, lncRNA, miRNA) which mediate cell-to-cell cooperation, immune reaction, inflammatory response, and reparation [41,42]. In addition, only a few studies have examined the functions and roles of exosomes derived from platelets. Janiszewski et al. successfully isolated exosomes from platelets and demonstrated their relation to the pathophysiology of sepsis [43]. In 2014, Torreggiani et al. isolated exosomes from PRP as novel effectors in human platelet activity [44]. They firstly discussed the use of PL-EXOs for bone tissue regeneration. Recent studies further underlie the beneficial effect of PL-EXOs from PRP in preventing osteonecrosis [45] and promoting the reepithelization of chronic wounds [37]. Based on these findings, it is relevant that PL-EXOs carry some of the most important GFs of platelet origin to protect them from destruction before arriving to the target cells. Moreover, PL-EVs can be easily isolated from activated PRP or platelet lysates principally by ultracentrifugation and ultrafiltration. Due to the composition of the surrounding membrane, their biological cargo is protected from rapid catabolism and processing by macrophages [46]. PL-EVs are free to move with body fluids, so they can also be regarded as nano-delivery treatments [47,48]. However, the number and final cargo of PL-EVs is mainly affected by the activation process with agonists or mediators, hemodynamic stress, ageing and inflammatory pathologies [49].

Recently, there has been great interest in the use of EVs from various cell types as therapeutic tools in human and veterinary medicine [50,51,52,53]. As concentrated platelets by means of PRP possess reparative and healing machinery, it is expected that PL-EVs might exhibit similar beneficial therapeutic properties under normal conditions due to their molecular cargo. They might have therapeutic potential as they can support coagulation, angiogenesis, regulate immunity and accelerate tissue repair. PL-EVs derived from resting platelets versus thrombin-activated platelets demonstrate mild or strong haemostatic properties, respectively [54,55]. Moreover, exosomal PL-EVs have been beneficial in the treatment of chronic injuries and trauma [56,57], alleviating knee osteoarthritis [58], promoting wound healing [37] or modulating the progression of osteonecrosis [45].

## 2. Platelet-Rich Plasma

In order to increase the chances of cure in the context of regenerative medicine techniques, the next approach based on platelet derivatives, such as PRP, has attracted the attention of researchers and clinicians. On the other hand, the lack of reproducibility—mainly due to non-standardized separation methods, platelet content, donor variability, storage conditions and activation protocol—impact the cell profile and secretory component of the final product and therefore hinder its subsequent clinical use. At least more than 20 different PRP production devices are currently available and are applied without standardization [59]. Studies also often report limited characterization of the content of PRP [60].

**Figure 1:**
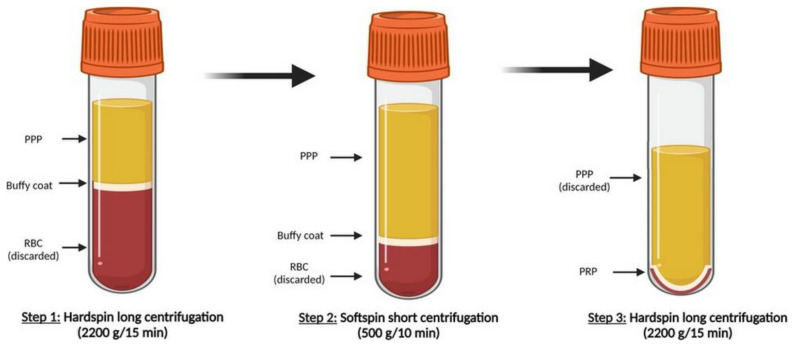
Schematic illustration of PRP preparation by three-step centrifugation method developed in our laboratory. (Created with BioRender.com). Abbreviations: RBC, red blood cells; PPP, platelet-poor plasma; PRP, platelet-rich plasma.

PRP is defined as a higher concentration of autologous human platelets exceeding physiological concentration in a small volume of plasma. There are many ways to prepare PRP with several procedural variables, such as the centrifugation parameter (speed, number of speeds), the volume and method of drawing blood and the type of anticoagulants which may affect platelet yield. Generally, PRP is obtained from whole blood supplemented with an anti-clotting agent after cycles of centrifugation. As it was described in our previous clinical study [61], our manual preparation of PRP (Figure 1) starts with a whole blood centrifugation, called “hard centrifugation”, in order to separate the blood into three layers: a lower layer with red blood cells (RBCs), buffy coat rich in white blood cells (WBCs) and platelets in an upper layer with plasma. Plasma in combination with buffy coat is collected and submitted to a further centrifugation, called “soft spin”, to separate WBCs. The third “hard centrifugation” is performed to obtain a fraction poor in platelets (upper layer) and a fraction rich in platelets (inferior layer). To take effect, PRP has to be activated to induce platelet degranulation, which releases more than 30 types of proteins from α-granules (e.g., TGF-β, PDGF, bFGF, EGF, VEGF, connective tissue growth factor—CTGF, IGF, IL-1, PF4). These proteins were found to promote the formation of extracellular matrix, cellular replication, angiogenesis, cell proliferation and differentiation [62]. Upon intra-articular injection of non-activated PRP into a defect, PRP can be activated by contact with local tissue factor [63,64].

Currently, numerous publications show that PRP is effective in the treatment of several disorders, including musculoskeletal pathologies, such as chronic sports-related injuries of the muscles and tendons, and degenerative joint diseases. In the context of orthopaedics, a series of previous studies on PRP treatment revealed its positive effect in patients with osteoarthritis compared with hyaluronic acid injection and placebo [65]. Various in vitro [66,67,68] and animal studies [69,70,71,72] also used PRP to demonstrate the beneficial effect of PRP on cell proliferation, structural modulation and inflammation status in the knee joint.

Despite their wide clinical use, the requirement for autologous platelets presents a limitation in its safety application, and the mechanism by which PRP produces improvements in the field of repair and regeneration remains subject to debate. Given that effective factors secreted by PRP can be encapsulated and enriched in PL-EVs, there is an assumption that these particles (MVs and EXOs) have a similar biological action to platelets and that the direct application of these PL-EVs has no side effect even with allogeneic application. Based on these findings, the important immune and inflammatory roles of platelets can be in part substituted and the regenerative properties can be improved by using PL-EVs instead of living platelets.

## 3. Platelet-Derived Extracellular Vesicles

To manage and control all their tasks, platelets release—in addition to cytokines, chemokines and proteins—EVs into the bloodstream, which make up more than 25% of all EVs in the circulating plasma [40]. Therefore, the functional relevance of EVs in blood plasma and their considerable role in the biological function of activated platelets have become an important new focus of the current research.

Extracellular vesicles were originally identified in platelet-free plasma and were shown to be rich in negatively charged phospholipids and to support coagulation [26]. Circulating PL-EVs in healthy individuals are released from activated platelets or from megakaryocytes; however, the latter ones have less contribution to inflammation and complement activation, so therefore are currently less of a focus in investigations [73]. Megakaryocyte-derived EVs are constitutively released from megakaryocytes in the bone marrow into the blood and are dominant in healthy individuals, while PL-EVs increase in conditions with enhanced platelet activation. Common detection markers for EVs contain numerous platelet-specific molecules, such as adhesion proteins (e.g., CD41, CD42a, CD31, fibrinogen, thrombospondin), platelet-specific glycoprotein (GP) IIb-IIIa, or proteins involved in coagulation (e.g., FVa, FVIII, TF) and growth factors (e.g., VEGF, PDGF, TGF-β1). PL-EVs generally express the platelet activation markers P-selectin (CD62P) and lysosome-associated membrane glycoprotein 1 (LAMP-1 or CD107a), while megakaryocyte-derived EVs do not [74]. Membranes of PL-EVs are enriched in free cholesterol and phospholipids. Both megakaryocytes and platelets release EVs that express tissue factor (TF) and phosphatidylserine (PS) [75,76]. Both PS and TF are able to initiate the extrinsic coagulation pathway involving active Factor VII. On the other hand, one study noted that many PL-EVs do not bind annexin V (suggesting they are phosphatidylserine negative) and have a higher expression of glycoprotein Ib [77]. In vitro study showed that PL-MVs can bind the anticoagulant protein S and support the activation of protein C that led to the anticoagulant effect [78].

EVs derived from activated platelets can mainly be divided into two populations. The first population is composed of larger vesicles (100 nm–1 μm) as microparticles (PL-MPs) generated by plasmatic membrane gemmation. The second population, named PL-EXOs, is composed of small vesicles (<100 nm) which are of endosomal origin and are released during the fusion of multivesicular bodies to the plasma membrane by the endocytic pathway. The basic characteristics of these two subtypes are summarized in Table 1. EVs are identified based on their size, density [24,79] and on the biochemical composition, including proteins, lipids, metabolites, and nucleic acids. Their proteomic profile is dynamic and the variation in EVs subtypes is likely to depend on the isolation and activation procedures. Markers that uniquely identify EV subtypes are almost lacking to date. Therefore, is difficult to distinguish between PL-MPs and PL-EXOs.

Usually, EVs contain markers which indicate their origin, e.g., CD41 for platelets [38], but the composition of surface markers of EVs may differ from parental cells because of differences in activation and formation. The endosomal sorting complex required for the transport (ESCRT) pathway is considered to be the most important mechanism of exosome formation [80,81], but sorting of exosomal cargo can also occur via an ESCRT-independent mechanism [82]. PL-MPs are produced mainly by budding of the plasma membrane [36]. The most frequently identified proteins of EVs recognized as exosomal markers are tetraspanins (e.g., CD9, CD63), tumour susceptibility gene 101 (TSG101), and programmed cell death 6–interacting protein (PDCD6IP or ALIX) [83]. The selective enrichment of CD63 in PL-EXOs may provide a clue for their possible extracellular function [28]. The study by Israels et al. has also shown that CD9 is associated with b3-integrins in both resting and stimulated platelets [84]. PL-EXOs contain various platelet plasma membrane markers that allow the identification of vesicles [28], such as membranous GPs that mainly include the platelet activation factors GPIIb/IIIa and GPIb/IX, adhesion molecules and integral proteins, among others [85]. Results of proteomic analyses also revealed the presence of HSP70, GPIb, GPV and WNT glycoproteins in PL-EXOs, which have a role in regulation of WNT signalling in monocytes and endothelial cells [30]. An increased amount of chemokines PF4 (CXCL4), pro-platelet basic protein (CXCL7) and cytoplasmic high-mobility group box 1 (HMGB1) protein was detected in exosomes after platelet activation with thrombin and collagen [86]. PL-MPs contain prothrombinase complex and the α-granule-derived factor V and X [87], suggesting their role in the coagulation process. Furthermore, PL-MPs express proteins including the fibrinogen receptor αIIb/β3, the von Willebrand factor, GPIb, P-selectin (CD62P) and platelet endothelial cell adhesion molecule-1 (PECAM-1 or CD31) [88,89]. The lipid raft protein, flotillin, is a frequently used marker of MPs [90]. In addition, two subtypes of PL-EVs can be distinguished for their RNA content in terms of RNA types and RNA amount. PL-EXOs are enriched in specific miRNAs, including miR126-3p, mi-R21, mi-223, miR-339, miR-328, miR-22, miR-185 and miR-320b, which may influence the behaviour of targeted cells and are associated with numerous human diseases [29,91,92,93,94].

**Figure 2:**
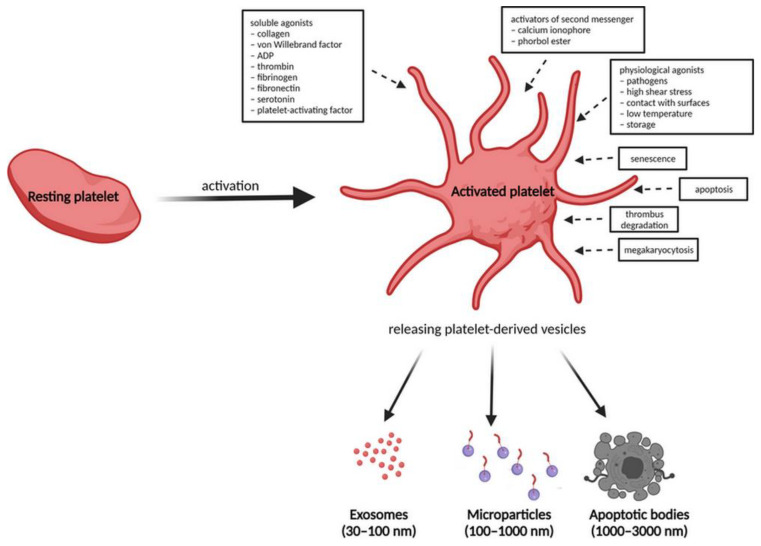
Activation process of anucleated platelets in the blood and release of multiple EVs as a result of morphological and functional changes. (Created with BioRender.com).

After activation, the outer membrane of microvesicles is invaded and released out of platelets through extracellular secretion. The dynamic content of PL-EVs including proteins from the platelet membrane, cytosol, organelles, adhesion receptors, coagulation factors, transcription factors, growth factors, active enzymes, cytokines, chemokines and their receptors is mainly dependent on the mechanism of platelet activation, the agonist used and the time of stimulation. Previous knowledge includes the formation of PL-EVs during the process of platelet activation by soluble agonists, activators of second messengers (such as calcium ionophores and phorbol esters), physiological agonists (pathogens, high shear stress, contact with surfaces, low temperature and storage) or during platelet senescence and apoptosis, thrombus degradation and during megakaryocytosis (Figure 2) [95,96]. Soluble agonists include collagen, von Willebrand factor, adenosine diphosphate (ADP), thrombin, fibrinogen, fibronectin, serotonin and platelet-activating factor [97,98].

Different processes of platelet activation may lead to the formation of heterogeneous PL-EV populations with different surface marker expression and protein profiles, which may affect their role in intercellular communication [99]. Several comparative studies have been performed, which compared the effect of different activators on platelet activation and the subsequent release of exosomes [49,95,100,101]. For example, EVs from platelets activated in vitro with ADP contain different protein cargo in comparison with those activated by collagen or collagen and thrombin [99], implying that these EVs could also have different functions.

### Isolation of Platelet-Derived Extracellular Vesicles

To date, there is no standardized method to isolate PL-EVs and there is no single detection technique with high sensitivity to detect all EVs from platelets or other biofluids. It is often impossible to compare the results of studies, as various isolation methods produce EVs with different yields and purities, and detection techniques mainly characterize a certain fraction of the total EV population. Therefore, the standardization of protocols is recommended to reduce variability in phenotyping, sizing and enumerating PL-EVs. The isolation process of EVs from plasma is complicated by the fact that plasma contains mitochondria and other non-EV particles beside platelets with similar sizes and densities (such as lipoproteins) and that plasma is of high viscosity.

The first protocols for blood collection and plasma preparation for studies of EVs were published in 2013 [102]. Later studies have shown that PL-EVs can be obtained through a four-step method: (1) PRP is prepared from whole blood; (2) platelets are further isolated from PRP; (3) platelets are activated to induce the release of EVs; (4) EVs are isolated by differential centrifugation [33]. However, because these steps are unwieldy, the agonists cannot be widely used in clinical practice and platelets may be prematurely activated to release exosomes during the separation and purification steps. Therefore, before clinical application, there is a need to standardize a rapid method for releasing high yield of pure exosomes after PRP activation by the use of common drugs (thrombin or calcium gluconate).

In addition, the concentration of PL-EVs and their cargo can be affected by many preanalytical and postanalytical factors, including mainly blood collection, handling, timing and storage [103,104]. The choice of anticoagulant is important for PL-EV characterization and quantification with an emphasis on minimizing in vitro platelet activation and consequent PL-EVs release. The most used anticoagulant that meets these criteria is sodium citrate [105]. It has been shown that other, frequently used anticoagulants, such as ethylenediaminetetraacetic acid (EDTA) or heparin, affect EV quantification. However, EDTA [106] is suitable when focusing on RNA analysis. Citrate–dextrose solution (ACD) alone is used when EVs derived from washed platelets are studied in vitro [107]. The combination of ACD and EDTA is an alternative anticoagulant for the quantification of circulating PL-EVs in plasma samples [108].

Another important consideration for the optimization of PL-EV quantity and quality is the storage of whole blood or platelet concentrates. Wisgrill et al. [109] confirmed that numbers of PL-EVs and their functionality are stable in sodium citrate for 8 h at room temperature. Additionally, PL-EV counts were stable for 48 h at room temperature in EDTA. Ex vivo resting platelets undergo platelet storage lesion, and they are subjected to necrotic or apoptotic changes, as well as to activation. Concentrations of PL-EVs are increasing in platelet concentrates during storage, suggesting that platelet aging is associated with the apoptotic release of EVs [110]. Freezing–thawing of platelet samples also leads to an increase in PL-EV numbers in samples, while long-term (>20 months) storage of samples at −80 °C has been reported to result in decreased PL-EV levels [111,112]. The mechanism underlying the release of EVs is likely to differ between the aging platelets and those activated by an agonist [113]. Since liquid PRP preparations only have a short half-life (~5 days) and are required to be kept at room temperature, frozen PL-EV preparations could be an attractive alternative [114,115]. Up to now, there is no optimum storage condition for isolated PL-EVs and their shelf-life, and the majority of published articles did not report any specific storage conditions. The most common way to store PL-EVs is at −80 °C [116]. In any case, it is necessary to determine storage conditions for both blood and PL-EVs to compare different approaches and evaluate the stability and functionality over time before their therapeutic use [117].

The most frequently used methods to isolate PL-EVs are based on ultracentrifugation, which is in fact a concentration method, and on size-exclusion chromatography (SEC). Ultracentrifugation can lead to the co-isolation of non-EV components such as protein aggregates and lipoproteins, leading to the disruption of EVs and resulting in variable recovery rates. On the other hand, SEC results in the purest EV fraction, with EVs >60 nm in diameter and separated from the bulk of high-density lipoproteins, cellular debris, protein aggregates, soluble proteins and very large proteins such as von Willebrand factor [116]. It has been shown that SEC does not result in aggregation and preserves the integrity and functionality of EVs better than ultracentrifugation [118], but we must not forget that SEC isolate all plasma EVs, including those derived from leukocytes and/or residual platelets, which may affect downstream analyses. In the absence of a common marker expressed by all EVs, it is impossible to isolate the total EV population. Based on the chosen antibody, immunocapture techniques with monoclonal antibodies and magnetic beads can be used to specifically isolate subpopulations of EVs on the basis of their immunophenotype. However, this technique may lead to nonspecific binding due to the cross-reactivity of the antibodies, interference with other ligands in plasma and difficulties in elution.

## 4. Therapeutic Use of Platelet-Derived Extracellular Vesicles

EVs from various cell types, mainly including mesenchymal stem cells (MSCs), are currently being explored as “cell-free” therapeutic tools and have been extensively studied for their role in stimulating tissue regeneration by conferring proangiogenic, proliferative, anti-apoptotic and anti-inflammatory actions thorough transport of their protein cargo and RNAs [119,120]. The growing number of recent studies also suggests that the regenerative potential of the MSC treatments is mediated by EVs or conditioned medium rather than by cells [121,122,123,124].

With the increased knowledge of cell-derived EVs, the potential of PL-EVs has also attracted increased attention in past years, as they represent the largest type of EVs in circulation. Due to the capacity of EVs to transfer proteins, lipids, metabolites and nucleic acids, PL-EVs may influence various important physiological and pathological functions via intracellular communication, including coagulation [125,126], immune response and inflammation [127,128], angiogenesis, wound healing [37,129,130] and carcinogenesis [131]. In view of these findings, PL-EVs might be not only an effective and safe alternative to PRP, but they may be useful for developing prospective therapeutic applications in haemostasis, tissue regeneration, immunomodulation and as drug-delivery vehicles.

The following in vitro and animal studies highlight the use of therapeutic PL-EVs that could become part of future strategies used for tissue healing. It is known that the main fraction of short RNAs is localized in PL-EVs, which protect them from degradation in circulation. Thus, these vesicles can be considered as biological nanovectors able to transfer specific signals and modulate gene expression in recipient cells and may determine vascular and tissue response in disease conditions associated with platelet activation. High levels of miR-142-3p detected in PL-EVs from activated platelets enhanced the endothelial cell proliferation/dysfunction via Bcl-2-associated transcription factor (BCLAF1) [132]. Another short RNA, miR223 derived from thrombin activated PL-EVs, can regulate the endothelial expression of two of its mRNA targets, FBXW7 and EFNA1 [133]. It has been established that miR223 is highly expressed in platelets and has a cardioprotective and anti-inflammatory role [134]. Gidlöf et al. revealed active packaging of miR-22 into EVs and its active depletion from platelets with increased activation and indicated a possible paracrine role of released platelet miR-320b on endothelial cell ICAM-1 expression [29]. The results of the study by Li et al. suggested that thrombin-activated PL-EXOs inhibit ICAM-1 expression during inflammation and this process is mediated by miRNA223 via the regulation of the NF-κB and MAPK pathways [135]. It is not surprising that PL-EVs play an important role in endothelial phenotype and function, as platelets themselves have an unambiguous role in modulating endothelial cell function in the context of the inflammatory response as well as in ischemia/reperfusion, sepsis and early atherosclerosis. Gambim et al. reported that PL-EVs collected from patients with septic shock directly induced the apoptosis of rabbit endothelial cells [136]. Their results also confirmed that in sepsis, the increased generation of NO and the presence of bacterial elements can trigger the release of PL-EXOs. In addition, a study by Jiao et al. [137] demonstrated that PL-EXOs are major mediators that induce neutrophil extracellular trap (NET) formation in septic shock. PL-EXOs containing HMGB1 and/or miRNA-induced NET formation through the modulation of the Akt/mTOR-related autophagy pathway. Targeting PL-EXOs as mediators of key features of sepsis may present a novel therapeutic strategy for septic shock. As a kind of immune cell, PL-EVs has an inherent affinity for inflammation sites, including atherosclerotic plaque sites, so they could be used for the delivery of anti-inflammatory agents. Recently, Ma et al. [138] showed that nano-sized MCC950-loaded PL-EVs could selectively bind multiple cell types such as macrophages and epithelial cells and have an efficient anti-inflammatory effect on atherosclerotic plaque after intravenous administration in an ApoE-KO mice model of atherosclerosis.

Due to the presence of negatively charged PS on the surface of PL-EVs, which promotes the aggregation of prothrombin complexes during coagulation, they may represent an alternative to widely used platelet concentrates in treating trauma patients with critical bleeding. Phosphatidylserine can activate coagulation factors II and X, hence triggering the coagulation cascade. In a rat model of uncontrolled bleeding, PL-EVs from non-activated washed platelets enhanced hemodynamic stability in comparison with fresh platelets by mitigating the development of ischemia and metabolic acidosis [55]. Their results also demonstrated in in vitro and in vivo mice models that PL-EVs have potent therapeutic effects on vascular permeability and haemostasis.

Based on available data, PL-EVs may play a dual role in inflammation, stimulating either pro- or anti-inflammatory effects depending on the expression of surface markers and microenvironment-affected cargo. However, studies demonstrating their pro-inflammatory properties are predominant. It was found that they contain proinflammatory cytokines such as interleukin IL-1, IL-6 and tumour necrosis factor alpha (TNF-α), and can induce leukocytes to release inflammatory cytokines such as interleukin IL-1β, IL-6, IL-17, interferon-gamma, TNF-α, monocyte chemoattractant protein-1 and matrix metalloproteinase MMP-6 and MMP-9, which promote endothelial inflammation, worsening vascular integrity and endothelial dysfunction [139]. It was demonstrated that platelets are capable of propagating their vesicles through joint-draining lymph in rheumatoid arthritis as a result of inflammation [140]. Recent findings also suggested that PL-EVs may infiltrate the bone marrow during inflammations and induce haematopoiesis by interaction with bone marrow cells and their functional reprogramming. Other data indicated that PL-EVs exert strong immunomodulatory activity on vascular smooth muscle cells, which is manifested by cytokine IL-6 production and leads to the stimulation of vascular remodelling [141]. Up to now, only a few studies have demonstrated that PL-EVs might suppress inflammation primarily by inhibiting cytokine release. For example, PL-EVs released by stored platelets modify macrophage and dendritic cell differentiation toward less reactive states [142]. Studies have shown differences among PL-EVs populations in the capacity to aggregate monocytes and induce neutrophil extracellular traps [143]. The ability of PL-EVs to regulate adaptive immunity was also described by inducing anti-inflammatory signalling in plasmacytoid dendritic cells [144] and inhibiting the differentiation of regulatory T cells into proinflammatory cells through a mechanism involving P-selectin [127]. Phagocytic function induced by PL-MPs demonstrated in several studies might be beneficial for the maintenance of blood quality and influence blood vessel function. These studies observed that MPs released from platelets polarized monocytes into the resident M2 subset macrophages with an increase in phagocytosis capacity [128,145].

It has been found that lipid components of PL-EVs are important in the mediation of enhanced proliferation, migration and tube formation capacity of human umbilical vein endothelial cells (HUVECs) treated with these microparticles [146]. Brill et al. showed that PL-EVs induce angiogenesis both in vitro and in vivo in a rat model and the injection of PL-EVs into the ischemic myocardium may improve the process of revascularization after chronic ischemia [147]. It was demonstrated that PL-EVs may be utilized for treatment following brain injury according to previous results describing their positive role in neuronal cell proliferation, survival and differentiation to form glia and neurons [148]. The administration of PL-EVs following stroke in a rat model led to increasing neural growth at the site of injury and to significantly improved behavioural deficits [57].

In a diabetic rat model, the presence of growth factors, mainly bFGF, PDGF-BB and TGF-β encapsulated in PL-EXOs, increased re-epithelialization and collagen synthesis, which led to faster wound closure compared to untreated wounds. Additionally, PRP-EXOs increased the proliferation and migration of human microvascular endothelial cells (HMEC-1) and fibroblasts to a greater extent than PRP [37]. Subsequently, Tao et al. demonstrated that PRP-EXOs successfully promote the re-epithelization of chronic cutaneous wounds via the activation of Yes-associated protein (YAP) in a diabetic rat model [45]. According to recent results by Iyer et al., the use of PRP-EXOs to facilitate soft tissue healing appears promising [56]. In this study, the effect of PRP-EXOs and bone marrow-derived MSC-EXOs was compared in a rat model of muscle injury. Both tested cell-free products facilitate recovery after a muscle strain injury due to the modulation of inflammation, fibrosis and myogenesis.

The application of PL-EVs might be an important step forward in the progress of osteoarthritis (OA) therapy. In the recent study by Liu et al., it was demonstrated that PRP-EXOs had a better effect in promoting chondrocyte proliferation and migration and attenuating apoptosis than PRP in a rabbit model in vitro and in vivo [58]. Their preliminary results also suggest that PRP-EXOs probably ameliorate OA via the Wnt/β-catenin signalling pathway, but it needs further research to illuminate the precise mechanism by which PRP-EXOs are involved in treating OA. In another work, the treatment of chondrocytes in vitro with PL-EVs enhanced the expression of anabolic markers such as type II collagen, SRY-box transcription factor 9 (SOX9) and aggrecan while preventing proinflammatory cytokine release compared to treatment with full blood product [149].

PL-EVs positively modulate the growth, migration and differentiation potential of stem cells from different sources. The enhanced potency of stem cell populations resident in tissues is important in preventing degenerative diseases. The first described role of EXOS derived from PRP in tissue regeneration was published in 2014 by Torreggiani et al. [44]. They demonstrated the potential effect of PRP-EXOs on the proliferation and migration of MSCs. In addition to bone marrow MSCs, PL-EVs increased the gene expression of human telomerase reverse transcriptase (hTERT) in umbilical cord-derived MSCs in vitro [150]. These results suggest that PL-EVs could potentially prolong the lifespan of MSCs, but this statement needs to be definitively verified in vivo.

Currently, three clinical trials involving PL-EVs or PL-EXOs are registered on www.clinicaltrials.gov (22 July 2021). One of these studies has been completed and two of them are recruiting/about to open to accrual. The trial NCT04281901 with results evaluated the efficacy of the autologous blood-derived product called platelet- and extracellular vesicle-rich plasma (PVRP) for the treatment of chronically inflamed post-surgical temporal bone cavities with 25 participants. The same group of researchers is currently monitoring the effect of PVRP for the treatment of chronic tympanic membrane perforations in chronic middle ear infections on 100 participants in clinical trial NCT04761562. Another trial, NCT04849429, is focused on the establishment of controlled, randomized and double-blind clinical trial to compare the safety and efficacy of PRP with exosomes in chronic lower back pain.

The lack of generally accepted standard methods for the determination, isolation, storage and quantification of the PL-EVs limits our efforts in understanding their biological role in the pathogenesis and therapeutic use of several diseases [116,151,152]. An ignorance of preanalytical factors, inefficient preparation or inadequate storage of platelets may lead to improper interpretations in further studies. Taking into account current knowledge about the diverse and sometimes contradictory functions of PL-EVs, achieving their full therapeutic potential will depend on the clear separation of PL-EVs subtypes and the careful development of best-practice standard protocols for PL-EVs generation and isolation.

## 5. Summary

The results of pre-clinical research are encouraging, as PL-EVs might be both the key part of the mechanism in tissue regeneration induced by blood derived products, especially PRP, and a potential alternative option instead of them. PRP has long been known to be effective in accelerating tissue repair, but the underlying mechanism is still not fully understood. The discovery and increased interest of PL-EVs has led to a more comprehensive and profound understanding. Although PL-EVs were identified as procoagulant particles released by activated platelets, today, it is known that these extracellular particles from anuclear platelets are capable of regulating the transcription, RNA stability, translation and metabolism of their target cells. There are numerous advantages in terms of biological importance, cost and efforts by substituting stem-cell-derived EVs by PL-EVs. In the case of PL-EVs, there is no requirement for a GMP facility for ex vivo cell expansion and there is no danger in concerns related to possible teratogenic risks. PL-EVs can be directly produced from collected platelet concentrates, in contrast to MSCs that require a step of isolation and ex vivo expansion with potential contamination by EVs from fetal bovine serum. Clinical-grade allogenic platelets are now easily accessible in many countries as a source of platelet lysate, therefore providing readily available resources for EV isolation. PL-EVs offer several advantages over the traditional platelet transfusion approach or therapeutic PRP, as they retain their functionality after undergoing freeze–thaw cycles, thus potentially eliminating the current limitations of storing, transporting and using fresh platelets within their short shelf-life.

Although the effects and underlying molecular mechanisms of PL-EVs in regeneration have not yet been elucidated, PL-EVs exhibit the capacity to act as an alternative option or even as an upgraded product of PRP. Identifying key molecular players and understanding the mechanisms of action will allow the rapid progression of PL-EVs to clinical development. Reaching their full therapeutic potential will depend on careful development of the best-practice protocols for their generation and isolation and clear standard separation of PL-EVs subtypes.

## Figures and Tables

**Table 1:** Characteristics of main platelet-derived extracellular vesicles.

	PL-EXOs	PL-MPs
Size of diameter	30–100 nm	100–1000 nm
Density	1.13–1.19 g/mL	1.25–1.30 g/mL
Sedimentation	100,000 g	10,000 g
Morphology	Cup-shaped, homogenous	Irregular shape, heterogeneous
Cell origin	multivesicular bodies	plasma membrane
Production mechanism	ESCRT–dependent, ESCRT–independent	direct budding from the plasma membrane
Lipids	Lipidic molecules from the parental cells (including BMP)	lipid content primarily derived from plasma membrane and resemble the parental cells (without BMP)
Surface markers	CD9, CD63, TSG101, ALIX	Flotilin
Platelet-specific proteins	CD31, CD41, CD42a, CD62P, PF4, GPIIb/IIIa, GPIb, GPV, CXCL7, HMGB1	Factor X, prothrombin, GPIb, TF, CD31, CD36, CD62P, CD61, CD40L, vWF, fibrinogen, thrombospondin
miRNA	miR126-3p, mi-R21, mi-223, miR-339, miR-328, miR-22, miR-185, miR-320b	

Abbreviations: ESCRT, endosomal sorting complex required for transport; BMP, bone morphogenetic protein; TSG101, tumour susceptibility gene 101; TF, tissue factor; PF4, platelet factor 4; HMGB1, high-mobility group box 1; vWF, von Willebrand factor; GP, glycoprotein.

## Data Availability

Not applicable.
